# Development and validation of a novel necroptosis-related gene signature for predicting prognosis and therapeutic response in Ewing sarcoma

**DOI:** 10.3389/fmed.2023.1239487

**Published:** 2023-08-17

**Authors:** Runhan Zhao, Yu Jiang, Jun Zhang, Yanran Huang, Chuang Xiong, Zenghui Zhao, Tianji Huang, Wei Liu, Nian Zhou, Zefang Li, Xiaoji Luo, Yongli Tang

**Affiliations:** ^1^Department of Orthopedics, The First Affiliated Hospital of Chongqing Medical University, Chongqing, China; ^2^Orthopedic Laboratory of Chongqing Medical University, Chongqing, China; ^3^School of Public Health, Chongqing Medical University, Chongqing, China; ^4^Department of Orthopedics, Qianjiang Central Hospital of Chongqing, Chongqing, China

**Keywords:** prognostic gene signature, Ewing sarcoma, random survival forest, necroptosis, immunotherapy, chemotherapy

## Abstract

Ewing sarcoma (ES) is the second most common malignant bone tumor in children and has a poor prognosis due to early metastasis and easy recurrence. Necroptosis is a newly discovered cell death method, and its critical role in tumor immunity and therapy has attracted widespread attention. Thus, the emergence of necroptosis may provide bright prospects for the treatment of ES and deserves our further study. Here, based on the random forest algorithm, we identified 6 key necroptosis-related genes (NRGs) and used them to construct an NRG signature with excellent predictive performance. Subsequent analysis showed that NRGs were closely associated with ES tumor immunity, and the signature was also good at predicting immunotherapy and chemotherapy response. Next, a comprehensive analysis of key genes showed that RIPK1, JAK1, and CHMP7 were potential therapeutic targets. The Cancer Dependency Map (DepMap) results showed that CHMP7 is associated with ES cell growth, and the Gene Set Cancer Analysis (GSCALite) results revealed that the JAK1 mutation frequency was the highest. The expression of 3 genes was all negatively correlated with methylation and positively with copy number variation (CNV). Finally, an accurate nomogram was constructed with this signature and clinical traits. In short, this study constructed an accurate prognostic signature and identified 3 novel therapeutic targets against ES.

## 1. Introduction

Ewing sarcoma (ES) is the second most frequent aggressive bone or soft tissue tumor in children and is characterized by early metastatic spread, a high recurrence rate, and a poor 5-year survival rate. Now, multimodal treatment of ES, including surgical resection, local radiotherapy, and intensive multi-agent chemotherapy, has been established (1). Despite comprehensive treatment, 30–40% of patients experience recurrence or metastasis (2). Furthermore, due to the lack of accurate prognostic statistical tools (3), diagnosing and treating ES patients remains difficult. Therefore, it is urgent for us to develop novel, effective treatment strategies and construct a reliable prognostic signature to improve the prognosis of ES patients.

Necroptosis is a caspase-independent and regulated cell death mechanism which primarily mediated by receptor-interacting kinase (RIPK) and mixed lineage kinase domain-like protein (MLKL) that has been connected to the formation and progression of many cancers and inflammatory illnesses (4–6). One study revealed that necroptosis might trigger and amplify antitumor immunity in malignancy immunotherapy (7). And another study also reported that induction of other cell death mechanisms, such as necroptosis, is gradually becoming a promising therapeutic strategy against malignancy (8). However, a study also reported that many cancer cells could induce endothelial necroptosis to facilitate extravasation and metastasis (9). These pieces of evidence suggest that necroptosis plays a complex role in cancer development. Thus, necroptosis-related genes (NRGs) may play an important role in the prognosis of ES. However, few studies have systematically used NRG signatures to predict the prognosis of ES patients and explored novel ES targets derived from NRGs. We conducted this research to address the aforementioned issue.

In the current study, we first developed a novel prognostic NRG signature to predict the prognosis of ES patients. Subsequent analyses revealed that the signature performed exceptionally well in terms of prognosis and immunotherapy and chemotherapy response. Meanwhile, 3 genes were identified as potential therapeutic targets and put into the Cancer Dependency Map (DepMap, https://depmap.org/portal/) and the Gene Set Cancer Analysis (GSCALite, http://bioinfo.life.hust.edu.cn/GSCA/) databases for further analysis. Finally, an accurate nomogram was also developed to assist clinicians in determining the prognosis of ES patients.

## 2. Materials and methods

### 2.1. Data collection and preprocessing

The train cohort (GSE17679 dataset) and validation cohort (GSE63157 dataset) were obtained from the Gene Expression Omnibus (GEO, www.ncbi.nlm.nih.gov/geo/). The list of NRGs was collected from several published articles (4, 7, 10) and the Kyoto Encyclopedia of Genes and Genomes (KEGG, https://www.kegg.jp) database. Both cohorts’ clinical data and NRGs expression matrix are located in Supplementary File 1.

### 2.2. Cell lines and cell culture

The human ES cell line (A673) and human bone marrow stroma cell line (HS5) were obtained from the America Type Culture Collection (ATCC, United States). And we cultured these cells in DMEM (HyClone; Cytiva) supplemented with 10% FBS (Shanghai ExCell Biology, Inc.) and 1% penicillin–streptomycin (100 IU/mL, Hyclone, Cytiva) in a humidified atmosphere with 5% CO2 at 37°C.

### 2.3. Construction of a prognostic NRG signature

Here, the univariate Cox analysis was used for preliminary screening, and NRGs with a value of *p* <0.05 were considered prognosis-related candidate genes. The random survival forest algorithm was then used to reduce the dimension of candidate genes by variable importance (VIMP) and minimal depth (MD). VIMP is a key index for random survival forest to evaluate the importance of variables, which is defined as the difference between the prediction error rate of the new model and the old model after replacing a variable with an arbitrary value, so the higher the VIMP value, the stronger the importance of the variable (11). MD is also a variable importance evaluation index. In the random forest, the closer the variable node is to the root node, the higher the value of the variable in the prediction, so the smaller the MD, the higher the variable importance (12). Finally, we utilized multivariate Cox analysis to screen out key genes from the top 10 genes in both VIMP and MD, and an optimal prognostic NRG signature was established based on key genes.

### 2.4. Validation of the prognostic NRG signature

Each patient was given a risk-score and divided into high-risk and low-risk groups based on the median risk-score. The calculation formula for risk-score is as follows (ho(t) is the model baseline risk coefficient, β is the risk coefficient of different genes calculated by the model, and X is the gene expression level):

$${}^{"}\mathrm{risk}-\;\mathrm{score}=\log{\left(h_{0}\left(t\right)*\exp\left(\beta_{1}X_{1}+\beta_{2}X_{2}+\ldots +\beta_{n}X_{n}\right)\right)}^{"}$$

Then, we analyzed differences in survival time, survival status, and expression of key genes among patients in different risk groups. Additionally, the Kaplan–Meier (K-M) survival analysis and the receiver operating characteristic (ROC) curve were performed to validate the accuracy of the signature. Finally, to evaluate the signature’s generalization ability, we used the other independent dataset, GSE63157, as validation data and followed the same procedure as described above.

### 2.5. Functional enrichment and GSEA analysis

To investigate the potential biological functions of NRGs in ES, we used the “clusterProfiler” R package (13) to perform Gene Ontology (GO) and KEGG enrichment analysis. Meanwhile, gene-set enrichment analysis (GSEA) was performed to investigate the difference in activated/inactivated biological pathways between the high- and low-risk groups.

### 2.6. Immunotherapeutic and chemotherapeutic response prediction

Immunotherapy has always been a hot topic in cancer treatment. Hence, we explored the potential of the signature in clinical immunotherapeutic response prediction. To explore the differences in immune microenvironment between high- and low-risk groups, we used the single-sample gene-set enrichment analysis (ssGSEA) algorithm to quantify the extent of infiltration of 28 immune cell species in each sample. The marker gene list was collected from a published article (14). Meanwhile, we explored the differences in the expression levels of eight recognized immune checkpoints between the high- and low-risk groups (15). To further investigate the difference in response to immunotherapy between distinct risk groups, one immunotherapeutic cohort from the “IMvigor210” R package, which investigated the efficacy of anti-PD-L1 antibody in patients with advanced urothelial cancer, was also included in our study as an external validation dataset.

As we know, chemotherapy is the most conventional treatment for ES. Hence, we also evaluated the predictive performance of the signature in terms of clinical response to 5 commonly used chemotherapeutic agents for ES. The “oncoPredict” is an R package (16) that predicts the half-maximal inhibitory concentration (IC50) of different chemotherapy drugs in each ES patient using the Cancer Therapeutics Response Portal (CTRP) and the Genomics of Drug Sensitivity in Cancer (GDSC) databases, allowing researchers to investigate the sensitivity differences of ES’s classic chemotherapeutics drugs in different risk groups.

### 2.7. Comprehensive analysis of key genes

To further explore these key genes, we performed a comprehensive analysis. First, we explore the difference in the expression level of each gene in tumor and normal tissue, as well as in the high- and low-risk group. Next, we separately performed correlation analysis between key genes, immune checkpoints, and clinical features, and the high prognosis value genes were performed for further analysis. Based on the median expression level of these genes, we divided the patients into high and low expression groups to perform a K-M analysis. Meanwhile, we explore whether these molecules are essential for ES cell line growth through the DepMap, a resource of genome-wide CRISPR-Cas9 knockout screens in hundreds of cancer cell lines. Finally, in order to further explore the function of these genes in pan-cancer, we utilized the GSCALite database (17) to perform genome-wide and pathway activity analysis. This web server is a tool for gene set cancer analysis and contains data from 33 types of cancers in the TCGA database, which provides researchers with a flexible way to analyze the complicated relationship between a gene set and single or multiple cancer types.

### 2.8. Real-time quantitative PCR (RT-qPCR)

In order to verify the accuracy of the analytical results, we carried out RT-qPCR experiments. Total RNA was isolated after 48 h of cell culture using the TRIzol (Invitrogen, United States) according to the manufacturer’s protocol. Reverse transcribed into cDNA using a reverse transcriptase kit (TaKaRa, Japan), and the expression levels of key genes were amplified by real-time fluorescence quantitative PCR (Bio-Rad, United States). GAPDH is the internal reference, with 2^-ΔΔ CT^ value indicating the relative expression level of target gene mRNA. All the primer sequences are shown in Table 1.

**Table 1:** Sequences of primers used in the present study.

Gene	Forward primer (5′-3′)	Reverse primer (5′-3′)
RIPK1	TGGGCGTCATCATAGAGGAAG	CGCCTTTTCCATGTAAGTAGCA
JAK1	CCACTACCGGATGAGGTTCTA	GGGTCTCGAATAGGAGCCAG
CHMP7	AAGCCTCTCAAGTGGACTCTT	ACAGACGATACACCTCCTCAG
GADPH	GGCTGCCCAGAACATCAT	CGGACACATTGGGGGTAG

### 2.9. Construction and evaluation of the nomogram

At the end of the study, we performed univariate and multivariate Cox regression analyses on the clinical data (including age, gender, and state) and risk-score to evaluate whether the signature could be utilized as an independent prognostic factor. Then, based on these variables, we constructed a predictive nomogram using the “rms” R package and evaluated the accuracy of the nomogram by creating a calibration curve.

### 2.10. Statistical analysis

Statistical analyses were conducted using R (version 4.03) and GraphPad Prism Software (version 8.2.1, United States). The student T-test and Wilcoxon signed-rank test were performed to compare group differences. K-M survival analysis was used to evaluate the survival differences between stratified patients. The area under the curve (AUC) was calculated from the ROC curve using the ‘timeROC’ R package. A value of *p* < 0.05 was considered statistically different unless otherwise specified.

## 3. Results

### 3.1. Construction of a prognostic NRG signature

**Figure 1:**
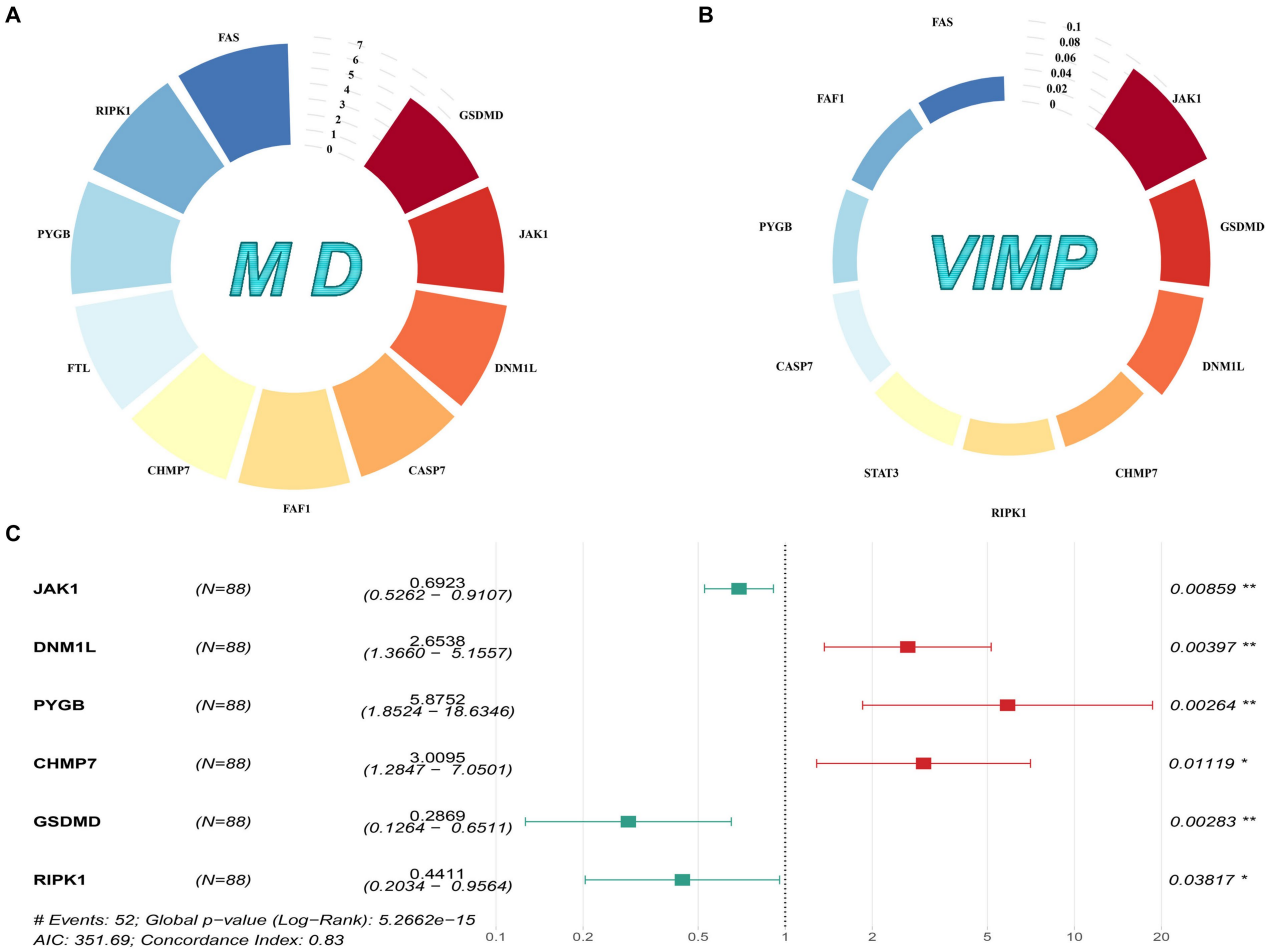
Gene selection and signature construction process. **(A)** The top 10 genes with MD values. **(B)** The top 10 genes with VIMP values. **(C)** The forest map of the multivariate Cox analysis on the 6 prognostic NRGs.

In total, 126 NRGs expression profiles were obtained from the GSE17679 and GSE63157 cohorts. Based on the univariate Cox analysis, 44 NRGs with a value of *p* < 0.05 were considered prognosis-related genes (Supplementary Figure S1). Then, we fed these NRGs into the random survival forest to compute the corresponding value of MD and VIMP. By performing cross-analysis between the top 10 genes of MD and the top 10 genes of VIMP, 9 NRGs were screened out (Figures 1A,B). Next, we subjected these 9 genes to multivariate Cox analysis, and 6 genes (JAK1, DNM1L, PYGB, CHMP7, GSDMD, and RIPK1) were identified as key genes. Finally, we used the 6 key genes to construct a valid prognostic signature (Figure 1C).

### 3.2. Validation of the prognostic NRG signature

**Figure 2:**
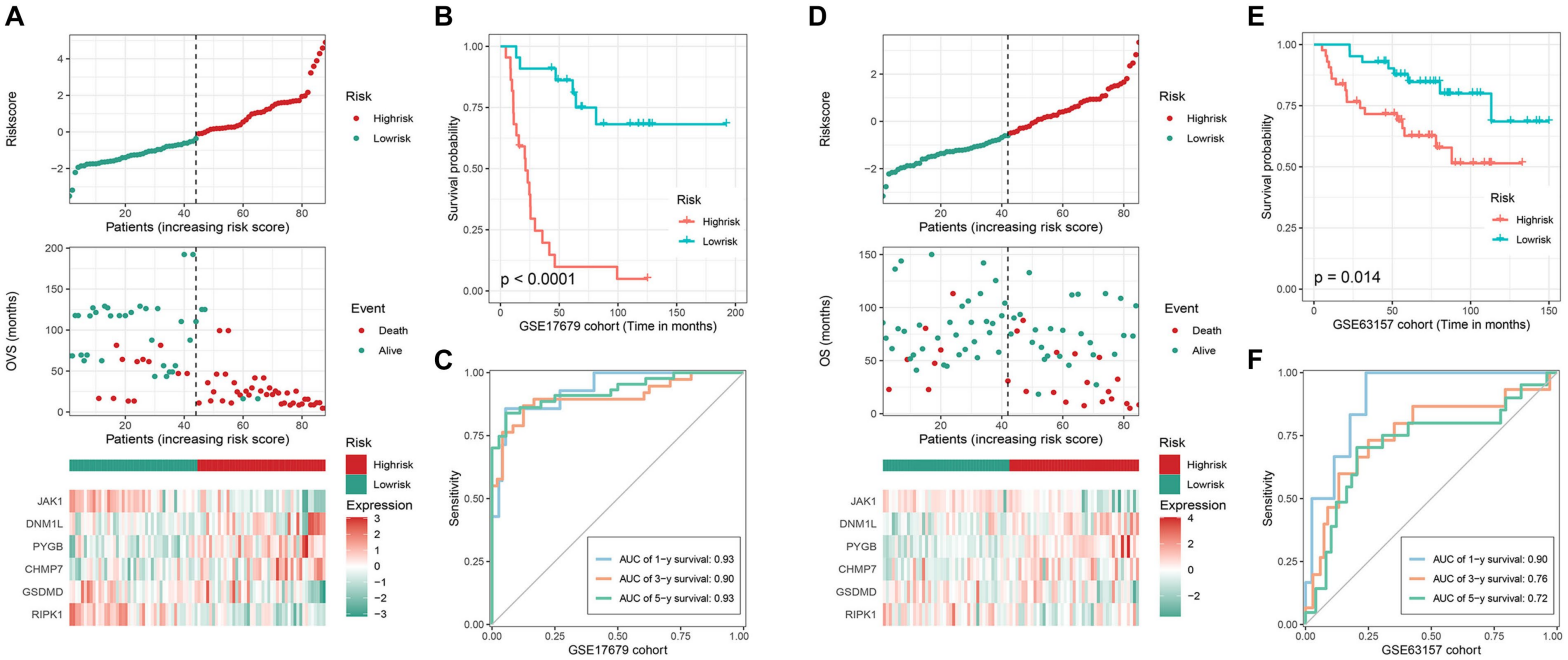
Validation of the efficacy of the signature. GSE17679 and GSE63157 cohort: **(A) (D)** the risk-score, survival status, and 6 key genes expression heatmap; **(B,E)** Kaplan–Meier survival analysis; **(C,F)** time-dependent ROC curves of the signature.

To validate the accuracy of the signature, we divided the patients into low- and high-risk groups based on the median risk-score. As shown in Figure 2A, the prognosis of the corresponding patient worsens as the risk-score rises, and there are also significant differences in the expression levels of key genes between the low- and high-risk groups. Meanwhile, the results of the K-M survival analysis (value of *p* <0.0001) showed that patients in the high-risk group owned worse survival time (Figure 2B) than those in the low-risk, and the AUC value of the ROC curve for 1, 3, and 5 years were 0.93, 0.90 and 0.93, respectively (Figure 2C). Additionally, the same trend was also observed in the GSE63157 cohort (Figure 2D). And as shown in Figures 2E,F, the K-M survival analysis (value of *p* = 0.014) and the AUC value of the ROC curve (1-year 0.90, 3-year 0.76, 5-year 0.72) were equally satisfactory. Therefore, all results demonstrate that this signature possesses excellent prediction ability.

### 3.3. Functional enrichment and GSEA analysis

**Figure 3:**
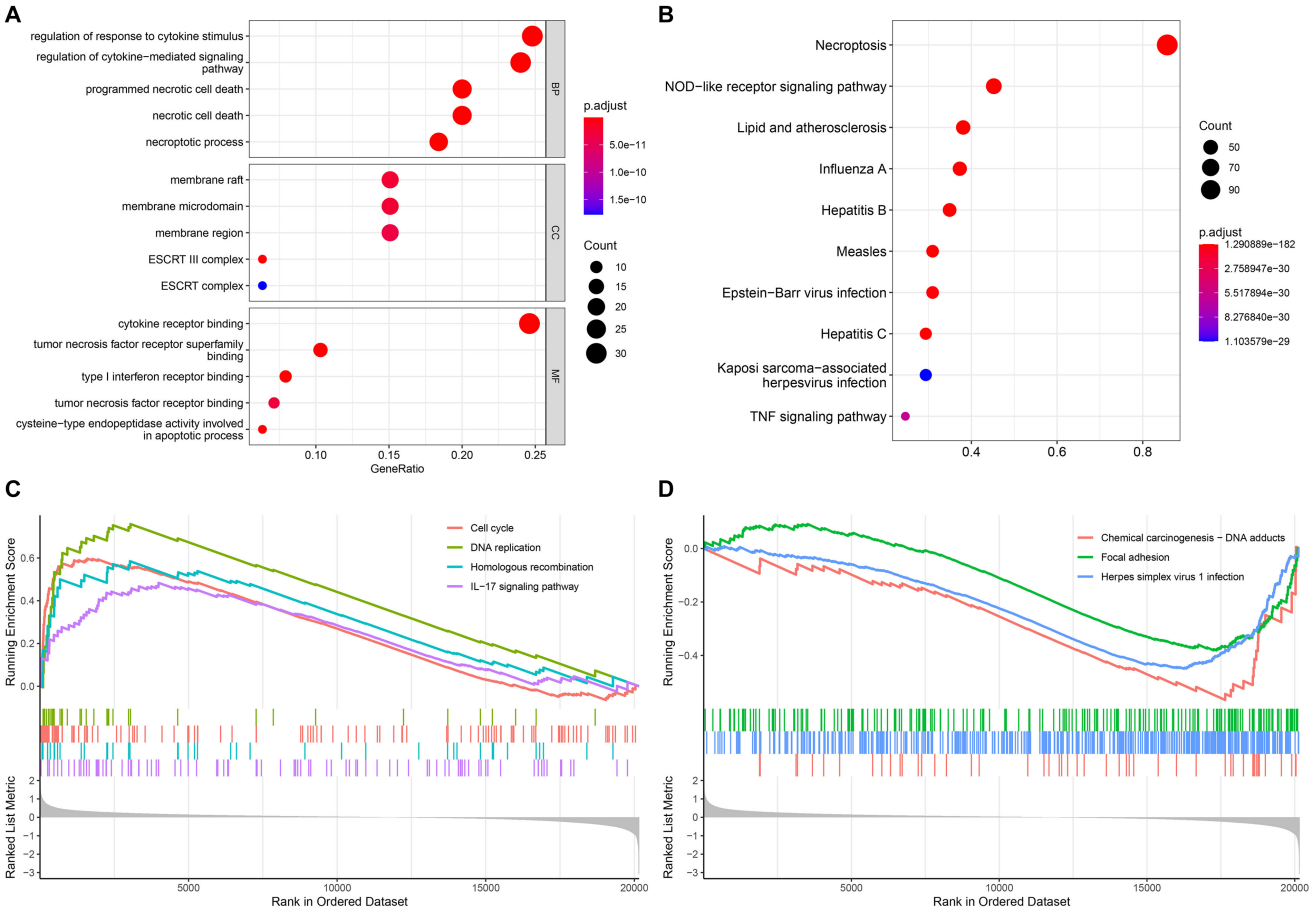
Functional enrichment and GSEA analysis. **(A)** Gene Ontology; **(B)** Kyoto Encyclopedia of Genes and Genomes; The GSEA enrichment reveal different activated signaling pathways in high-risk group **(C)** and low-risk group **(D)**.

The GO and KEGG analysis results are shown in Figures 3A,B. And we also found that NRGs enriched in some immune-related signaling pathways, such as PD-L1 expression and PD-1 checkpoint pathway in cancer, T cell receptor signaling pathway, and B cell receptor signaling pathway (Supplementary Table S1). Furthermore, the GSEA analysis revealed that the IL-17 signaling pathway, cell cycle, DNA replication, and homologous recombination were significantly enriched (value of *p* <0.05) in the high-risk group (Figure 3C), while the focal adhesion, chemical carcinogenesis - DNA adducts, and herpes simplex virus 1 infection were significantly enriched (value of p <0.05) in the low-risk group (Figure 3D).

### 3.4. Immunotherapeutic and chemotherapeutic response prediction

**Figure 4:**
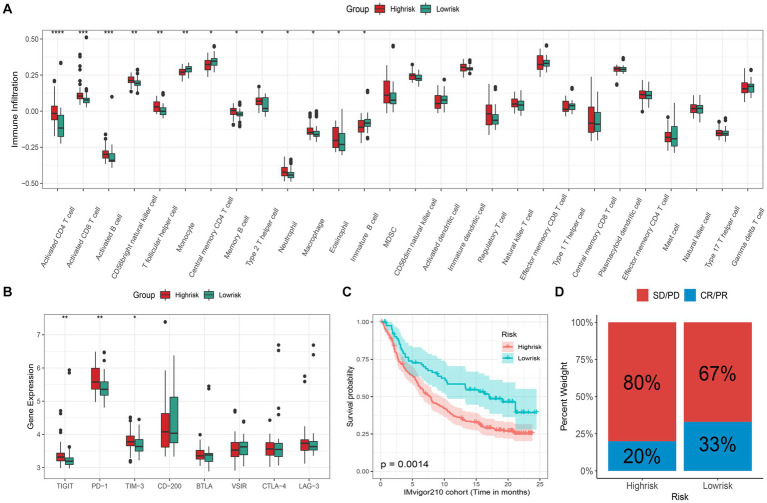
Immunotherapeutic response prediction. **(A)** Different infiltrating abundances of 28 immune cell types between high/low-risk group (^*^p < 0.05; ^**^p < 0.01; ^***^p < 0.001; ****p < 0.0001); **(B)** Different expression level of 8 immune checkpoints between subgroups; **(C)** Kaplan–Meier analysis of patients in the IMvigor210 cohort between high- and low-risk groups, and **(D)** the proportion of response to anti-PD-L1 immunotherapy between high- and low-risk groups (CR: complete response; PR: partial response; SD: stable disease; PD: progressive disease).

Many studies have discovered that necroptosis is related to tumor immunity, and the functional enrichment analysis results of our study also support this. Therefore, the application potential of the signature in immunotherapy deserves further study. Here, we first investigated the differences in immune cell infiltration patterns between the high- and low-risk groups. As shown in Figure 4A, ten kinds of immune cells were significantly higher infiltration in the high-risk group, including type 2 T helper cell and macrophage, which were related to immunotherapy resistance. And 3 types of immune cells were significantly higher in the low-risk group than in the high-risk group. Meanwhile, we also found that 9 kinds of immune cell infiltrating degrees were significantly correlated with risk-score (Supplementary Figure S2). In addition, the expression levels of 3 (TIGIT, PD-1, and TIM-3) immune checkpoints were significantly higher in the high-risk group than those in the low-risk group (Figure 4B). The above results have shown that there may exist differences in response to immunotherapy for patients in distinct subgroups. Therefore, the IMvigor210 cohort receiving anti-PD-L1 immunotherapy was utilized in the current study to validate the signature’s ability to predict patients’ immunotherapeutic response. We found that, in the IMvigor210 cohort, the prognosis of patients in the low-risk group is significantly better than that of the patients in the high-risk group (Figure 4C). Meanwhile, the low-risk group patients’ clinical response to PD-L1 blockade therapy was also significantly better than that in the high-risk group (Figure 4D). Hence, patients in the high-risk group may had poorer immunotherapy effects than those in the low-risk group.

**Figure 5:**
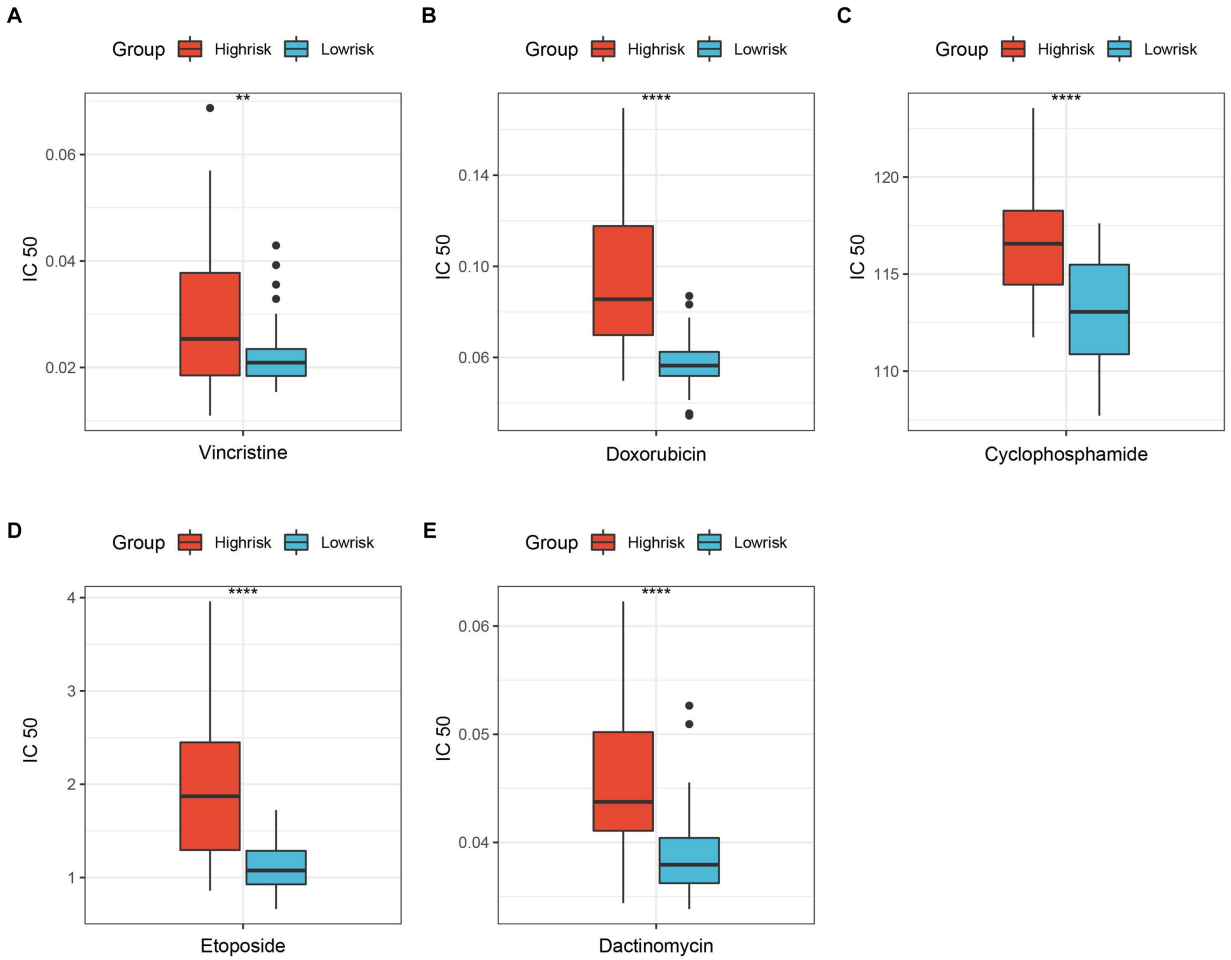
Differentially sensitive of ES’s classic chemotherapeutic drugs between high-risk group and low-risk group. **(A)** Vincristine; **(B)** Doxorubicin; **(C)** Cyclophosphamide; **(D)** Etoposide; **(E)** Dactinomycin. **p < 0.01; ****p < 0.0001.

Improving the efficacy of chemotherapy and avoiding severe drug toxicity has always been a major goal of researchers. The “oncoPredict” results showed that 5 common drugs were more sensitive to ES patients in the low-risk group than those in the high-risk group (Figure 5). Hence, patients in the low-risk group are more likely to benefit from chemotherapy.

### 3.5. Comprehensive analysis of key genes

**Figure 6:**
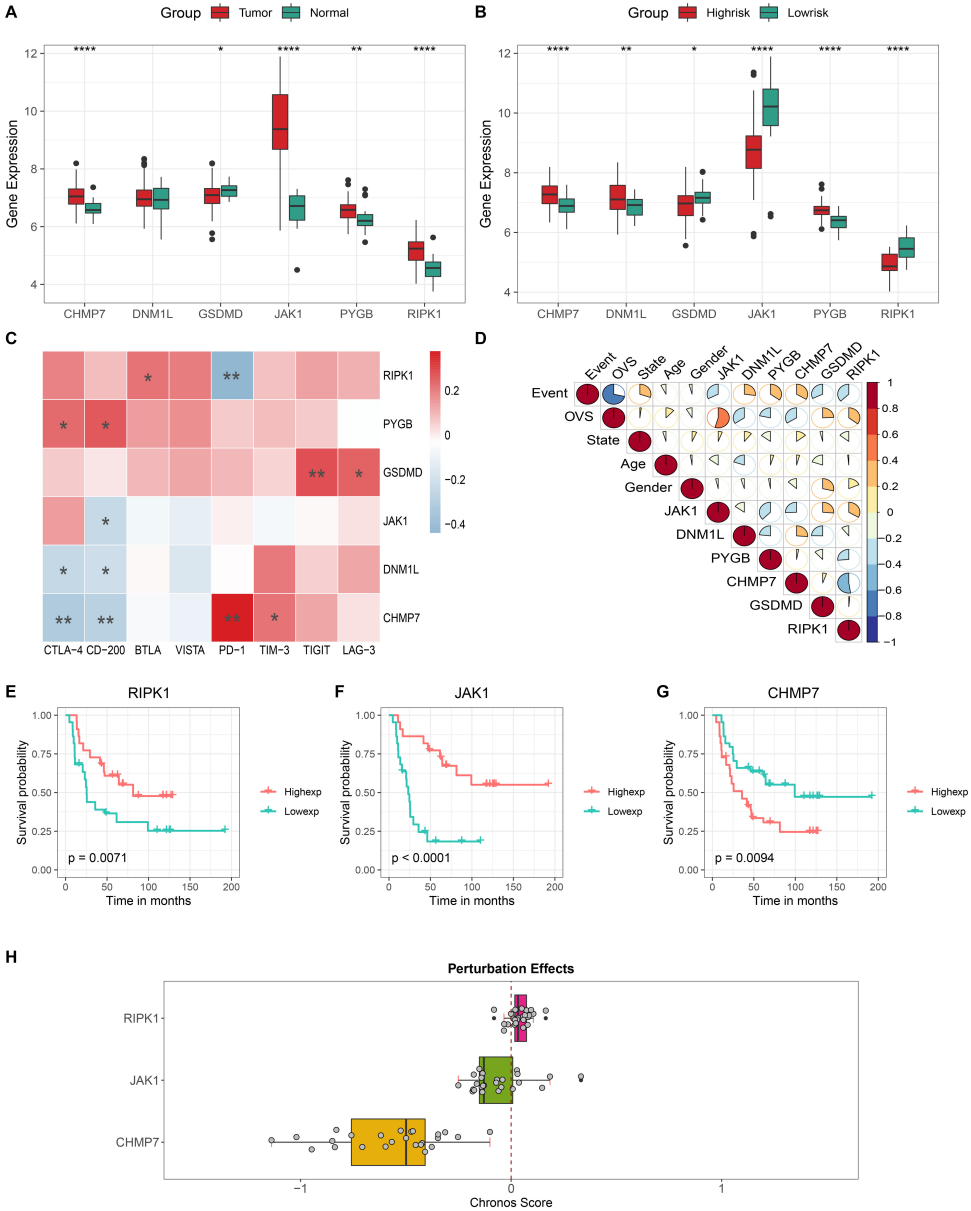
Comprehensive analysis of key genes. Different expression level of 6 key genes in the tumor/normal tissue **(A)** and high- / low-risk group **(B)**. Correlation analysis of the expression levels of 6 key genes with immune checkpoints **(C)** and clinical features **(D)**. Kaplan–Meier curves of patients with high and low expression of **(E)** RIPK1, **(F)** JAK1 and **(G)** CHMP7. **(H)** The boxplot shows the Chronos dependency score of 3 genes (a score of 0 indicates a gene is not essential and a lower Chronos score indicates a higher likelihood the gene is essential in a cell line).

The different expressions of 6 key genes between tumor and normal tissues or between distinct risk groups are shown in Figures 6A,B. The correlation analysis revealed that several immune checkpoints are closely associated with key genes (Figure 6C). In addition, we also found that 3 genes (RIPK1, JAK1, and CHMP7) showed a strong correlation with both OVS (overall vital survival) and event (Figure 6D), and the results of 3 genes’ K-M analysis were also satisfactory (Figures 6EG). Meanwhile, the DepMap results revealed that CHMP7 plays an essential role in ES cell growth (Figure 6H).

**Figure 7:**
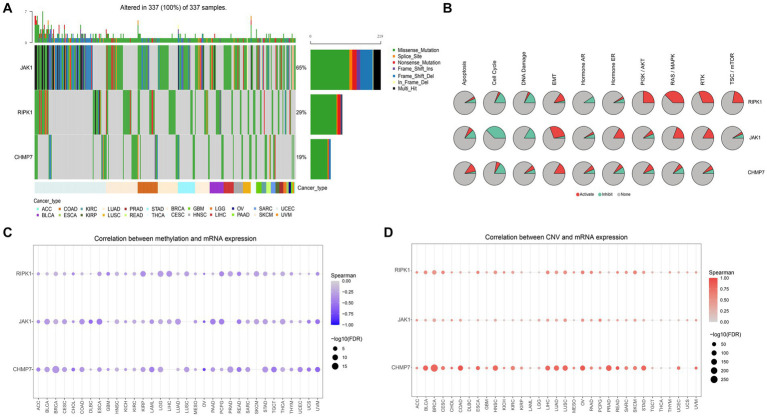
Genome-wide and pathway activity analysis. **(A)** Waterfall plot, shows the mutation frequency of 3 genes in 33 types of cancers. **(B)** The global activity of 3 genes in 33 types of cancers (the degree to which 3 genes contribute to activation/inhibition of 10 tumor-related pathways in 33 types of cancers). **(C)** The relation between methylation and gene expression. **(D)** The relation between CNV and gene expression.

Then the in-depth analysis results of the 3 genes by the GSCALite. The results of the genome-wide analysis revealed that JAK1 had the highest mutation frequency, followed by RIPK1, and CHMP7 had the lowest (Figure 7A). Meanwhile, the pathway activity study discovered that RIPK1 was significantly associated with RAS / MAPK and RTK pathway activation, while JAK1 was significantly associated with cell cycle pathway inhibition (Figure 7B). In addition, as shown in Figures 7C,D, we discovered that the mRNA expression of all 3 genes was negatively correlated with methylation expression and positively correlated with the percentage of copy number variation (CNV). Based on the above findings, 3 genes will probably become novel therapeutic targets for ES.

### 3.6. Validation of expression level of three key genes in ES cell

**Figure 8:**
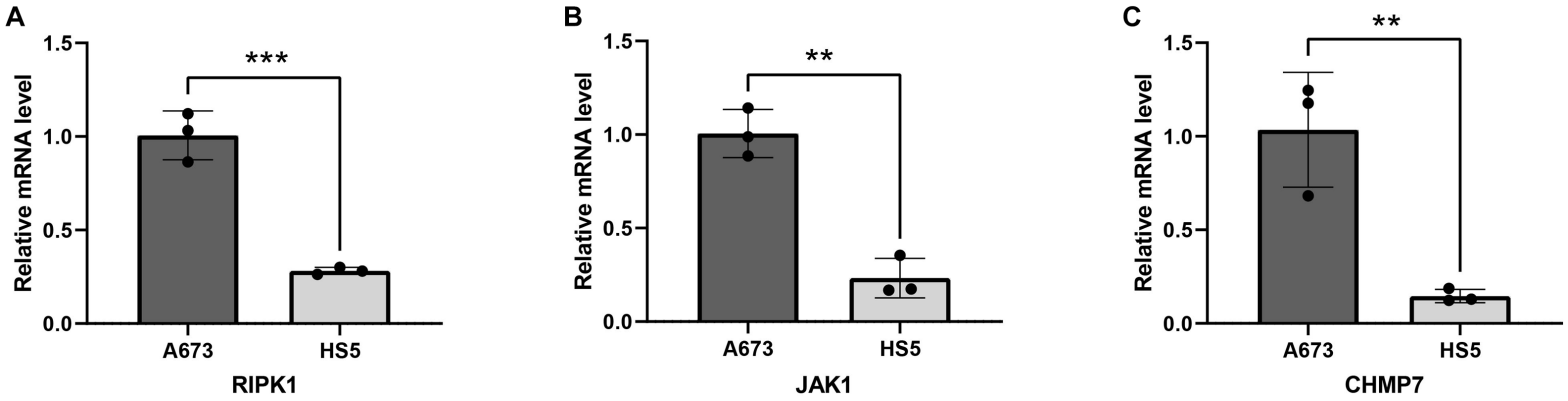
Validation of mRNA expression of 3 key genes in cell lines. **(A)** RIPK1; **(B)** JAK1; **(C)** CHMP7. **p < 0.01; ***p < 0.001.

The above analysis showed that RIPK1, JAK1, and CHMP7 are all significantly highly expressed in tumor tissue compared to normal tissue. To verify the accuracy of the analysis in this study, we performed RT-qPCR to compare the expression levels of 3 key genes between bone marrow stroma and ES cells. As shown in Figures 8A–C, the expression of RIPK1, JAK1, and CHMP7 in the A673 cell line was significantly higher than in the HS5 cell. Therefore, this cell experiment further verified the reliability of the results of this bioinformatics analysis.

### 3.7. Construction and evaluation of the nomogram

**Figure 9:**
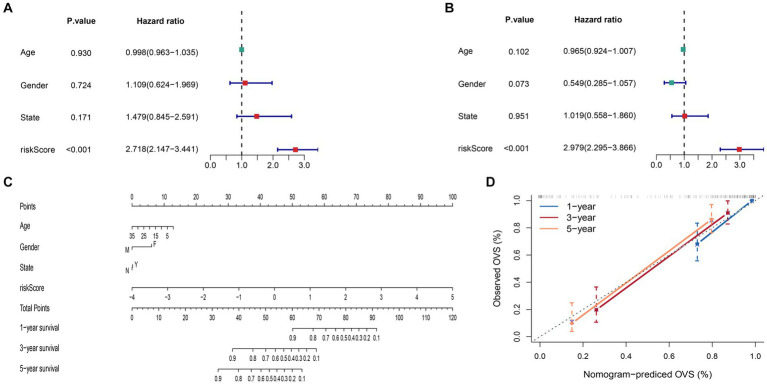
Construction and evaluation of the nomogram. **(A)** Univariate Cox regression analysis and **(B)** multivariate Cox regression analysis. **(C)** The nomogram constructed to predict the probability of patient mortality (State: N means progression no; Y means progression yes). **(D)** The calibration plot of nomograms between predicted and observed 1-, 3- and 5-year outcomes.

We discovered that the risk-score has significant significance in both univariate and multivariate Cox regression analyses at the end of this study, proving that the signature is a reliable, independent prognostic factor (Figures 9A,B). Then, we built a nomogram to predict 1-, 3-, and 5-year survival time of ES patients (Figure 9C). And, according to calibration plots, we found that the mortality estimated by the nomogram was close to the actual mortality (Figure 9D), confirming the nomogram’s excellent accuracy.

## 4. Discussion

Systematic treatments are the routine or even the only choice for cancer patients. Chemotherapeutics and target therapeutics are common systemic treatments, but treatment failure and side effects are frequently reported (18). Hence, there is an urgent need to find a new exploitable and safe systemic treatment mode. The emergence of tumor immunotherapy has greatly transformed the cancer treatment landscape. However, immune resistance has always been a challenge in immunotherapy. In most cancers, only one-third of patients respond to immune checkpoint inhibitors. In addition, resisting programmed cell death and the tumor lacking preexisting immunity can also cause this phenomenon (8, 19, 20). Thus, researchers were increasingly interested in inducing other cell death mechanisms or developing novel immunotherapy targets to treat cancers.

Necroptosis, a novel mechanism of programmed cell death, was discovered just over a decade ago (4). And with its crucial role in inflammation, necroptosis has attracted widespread attention from researchers. An article reported that necroptosis could enhance antitumor immunity by activating RIPK1/3 within the tumor microenvironment (TME). And in another study, a necroptotic cancer cell-mimicry nano-vaccine potentiated antitumor immunity in mice by promoting the expansion of NKG2D+ natural killer cells and CD8+ T cells (8). Based on the double attribute of necroptosis (novel cell death mechanism and the important role in the immune), we may find a novel way to improve the prognosis of ES.

In the present study, to lower the dimension of 126 NRGs in the current study, we used the univariate Cox analysis, random survival forest method, and multivariate Cox analysis. Finally, 6 key genes (JAK1, DNM1L, PYGB, CHMP7, GSDMD, and RIPK1) were identified to construct the prognostic signature. Here, the reason why we adopted the random survival forest algorithm rather than LASSO regression analysis is as follows: First, VIMP and MD, as two tested quantitative indicators, could be used for key gene identification. Second, it can balance errors within unbalanced data sets (11).

Previous studies have demonstrated the role of the immune microenvironment in tumor biology (21–24). The functional enrichment and GSEA analysis results in the current study also showed that NRGs were significantly linked to various immune-related pathways. Hence, we performed a comprehensive immune-related analysis to assess the potential of the signature for application in immunotherapy. The results indicated that there were substantial differences in the degree of immune cell infiltration among ES patients from different risk groups, and 8 immunological checkpoints were also differentially expressed in different ones. Furthermore, according to the IMvigor210 cohort, we found that low-risk patients showed a significant therapeutic advantage with stronger clinical responses and longer survival compared with high-risk patients. These results demonstrate the great potential of this signature in predicting clinical immunotherapy response. Surprisingly, the signature also performed well in predicting clinical chemotherapy response. Consequently, the signature is also beneficial for formulating chemotherapy and immunotherapy treatment strategies.

In order to further explore the influence of 6 key genes on the prognosis of ES, we performed a comprehensive analysis. And results showed that all genes are associated with prognosis and immune checkpoints. The 3 genes (RIPK1, JAK1, and CHMP7) play a more critical role in the occurrence and development of ES and maybe the potential targets for ES. Subsequent analysis confirmed the hypothesis. The DepMap results showed that CHMP7 is critical for the growth of ES cells. Moreover, the GSCALite results showed that JAK1’s mutation frequency was the highest, JAK1 was significantly associated with cell cycle pathway inhibition, RIPK1 was significantly associated with RAS / MAPK and RTK pathway activation, and all 3 genes were negatively correlated with methylation. Meanwhile, we also found that 3 genes (especially CHMP7) were all positively correlated with CNV, suggesting that CNV regulated their expression to some extent. Meanwhile, cell experiments also confirmed the accuracy of the analysis results. Hence, the 3 genes deserve in-depth exploration.

Receptor-interacting kinase 1 (RIPK1) is an important drug target not only due to its vital role in controlling the balance between cell survival and cell programmed death (apoptosis and necroptosis) (4, 10, 25) but also because its molecular structure is highly amenable for developing specific pharmacological small-molecule inhibitors (26). Numerous studies have shown that RIPK1 inhibitors present a potential therapeutic alternative for the management of a wide variety of inflammatory and degenerative diseases in humans, including colitis, dermatitis, traumatic brain injury, amyotrophic lateral sclerosis (ALS), multiple sclerosis (MS), etc. (5). However, the prognosis of different types of cancer is affected differently by RIPK1 (27). Low expression of RIPK1 is linked to a bad prognosis in head and neck squamous cell carcinoma and liver cancer (28, 29), whereas high expression of RIPK1 is linked to a poor prognosis in breast cancer (BC) and glioblastoma (30, 31). In the present study, we found that ES patients with higher levels of RIPK1 expression typically survive longer than those with lower levels. Coupled with the pivotal role of RIPK1 in necroptosis. Therefore, this gene is likely to be a therapeutic target for ES.

Janus kinases are a family of non-receptor tyrosine kinases whose members include TYK2, JAK1, JAK2, and JAK3 (32). Studies have revealed that a number of illnesses, including malignant tumors, involve the JAK/STAT pathway. This pathway suppresses antitumor immunity while promoting tumor survival, angiogenesis, and metabolism (33, 34). Moreover, among the JAK family kinases, JAK1 is a major driver of STAT3 phosphorylation (35). In the present study, we found that JAK1 was significantly associated with prognosis in ES patients, and genome-wide analysis also showed that JAK1 was significantly associated with inhibiting the cell cycle pathway. This finding suggests that JAK1 may be a potential therapeutic target for ES.

Charged multivesicular body protein 7 (CHMP7) is a critical member of the endosomal sorting complex required for transport (ESCRT) system and plays a crucial role in the endosomal sorting process pathway (36–38). ESCRT system is essential molecular machinery for sorting membrane proteins in eukaryotic cells, and its primary function is to promote the degradation of ubiquitin-tagged membrane proteins (39–41). CHMP7 and ESCRTIII can form a complex to jointly complete the contraction process, shear bud neck, and final membrane shedding (37). Therefore, aberrant CHMP7 expression in a variety of tissues can result in ESCRT system dysfunction, which in turn causes impaired protein degradation and consequent disease. A pan-cancer study shows that CHMP7 is low expressed in most tumor tissues, and patients with low expression have a poor prognosis (42). Nevertheless, in our study, the result is just the opposite. CHMP7 is highly expressed in ES tumor tissues when compared to normal tissues, and the high-expressing group has a poor prognosis. The results were also verified in subsequent cell experiments. Meanwhile, the DepMap results also revealed that CHMP7 is related to ES cell growth. Hence, these results enlighten us that CHMP7 plays a unique role in ES, which is worthy of our in-depth study.

Our study has significant clinical application value, and the constructed signature can be used as a reliable tool for predicting the prognosis of ES patients as well as helpful for the formulation of immunotherapy and chemotherapy strategies. Meanwhile, 3 potential therapeutic targets were identified, providing new treatment options for ES. The transformation of “cold tumors” into “hot tumors” has become an important direction of tumor research. Consequently, targeting these 3 key molecules to alter the infiltration of immune cells and high expression of immune checkpoints may help develop new immune combinations or novel immunotherapy drugs and promote personalized tumor immunotherapy.

Of course, our research also has some limitations. First, although we used different datasets to verify the accuracy of the signature, it is still a retrospective study in nature and is susceptible to the inherent biases of this research paradigm. Secondly, due to the bias in the sequencing results measured by different sequencing platforms, it is difficult to define the absolute threshold in the clinic. Finally, the PCR experiment of 3 key genes could be considered an external validation in a sense, but we only used one ES cell line, which is also a limitation of our study. Finally, we only conducted PCR experiments on 3 key genes and did not conduct more experiments for in-depth verification. This is also the limitation of our research and needs to be explored in depth in follow-up research.

## 5. Conclusion

In conclusion, our study constructed a reliable signature for predicting ES patients’ prognosis and therapeutic response. The subsequent analysis also screened out 3 potential therapeutic targets against ES, providing new options for treating ES patients. The nomogram constructed at the end of the study can help clinicians comprehensively evaluate the survival time of patients.

## Data availability statement

The original contributions presented in the study are included in the article/Supplementary materials, further inquiries can be directed to the corresponding authors.

## Ethics statement

Ethical approval was not required for the studies on humans in accordance with the local legislation and institutional requirements because only commercially available established cell lines were used. The manuscript presents research on animals that do not require ethical approval for their study.

## Author contributions

Conception and designation, data analysis, visualization were performed by RZ and YJ. Material preparation was performed by JZ and YH. Data collection was performed by RZ, YJ, JZ, YH, CX, ZZ, TH, WL, and NZ. ZL, XL, and YT guided the methodology section. The manuscript was written by RZ and reviewed by ZL, XL, and YT. All authors contributed to the article and approved the submitted version.

## Funding

This research was supported by National Natural Science Foundation of China (Grant No. 81873998) and Chongqing Young and Middle-aged Medical High-end Talent Studio (Grant No. cstc2022ycjh-bgzxm0103).

## Acknowledgments

The authors would like to thank GEO (https://www.ncbi.nlm.nih.gov/geo/), DepMap (https://depmap.org/portal/), and GSCALite (http://bioinfo.life.hust.edu.cn/GSCA) database for providing the data.

## Conflict of interest

The authors declare that the research was conducted in the absence of any commercial or financial relationships that could be construed as a potential conflict of interest.

The reviewer SL declared a shared parent affiliation with the authors to the handling editor at the time of review.

## Publisher’s note

All claims expressed in this article are solely those of the authors and do not necessarily represent those of their affiliated organizations, or those of the publisher, the editors and the reviewers. Any product that may be evaluated in this article, or claim that may be made by its manufacturer, is not guaranteed or endorsed by the publisher.
