# Supplementary material for: Development and validation of a novel necroptosis-related gene signature for predicting prognosis and therapeutic response in Ewing sarcoma
Source: Front Med (Lausanne). 2023 Aug 17;10:1239487. doi: 10.3389/fmed.2023.1239487 (PMC10470467; doi:10.3389/fmed.2023.1239487)
Supplement: Supplementary file 1 [file Data_Sheet_1.zip › supplementary material files/Supplementary Figure Legend.docx]

**Supplementary Figure S1.** 44 prognosis-related NRGs selected by univariate Cox analysis with p-value < 0.05.

**Supplementary Figure S2.** The correlation between risks-core and 9 immune cell infiltration (Blue, negative correlation; Red, positive correlation).
