# Supplementary material for: Development and validation of a novel necroptosis-related gene signature for predicting prognosis and therapeutic response in Ewing sarcoma
Source: Front Med (Lausanne). 2023 Aug 17;10:1239487. doi: 10.3389/fmed.2023.1239487 (PMC10470467; doi:10.3389/fmed.2023.1239487)
Supplement: Supplementary file 1 [file Data_Sheet_1.zip › supplementary material files/Supplementary Table S1.docx]

**Supplementary Table S1.** Immune-related signaling pathways in KEGG results.

| **ID** | **Description** | **Genes** | **-log10(P)** |
| --- | --- | --- | --- |
| hsa05235 | PD-L1 expression and PD-1 checkpoint pathway in cancer | CHUK,IKBKB,IKBKG,TICAM1,MAPK1,  MAPK14,IFNG,IFNGR1,IFNGR2,JAK1,  JAK2,STAT1,STAT3,TLR4 | 10.19 |
| hsa04660 | T cell receptor signaling pathway | MAP3K7,CHUK,IKBKB,IKBKG,TNF,MAPK1,MAPK8,MAPK14,MAPK9,IFNG | 5.36 |
| hsa04662 | B cell receptor signaling pathway | CHUK,IKBKB,IKBKG,MAPK1 | 1.42 |
| hsa04657 | IL-17 signaling pathway | TRADD,FADD,CASP8,TRAF2,TAB3,MAP3K7,CHUK,IKBKB,IKBKG,CASP3,TNF,MAPK1,MAPK8,MAPK14,TRAF5,MAPK9,IL1B,IFNG,HSP90AB1,TNFAIP3 | 17.13 |
