# Supplementary figures and images for: Development and validation of a novel necroptosis-related gene signature for predicting prognosis and therapeutic response in Ewing sarcoma
Source: Front Med (Lausanne). 2023 Aug 17;10:1239487. doi: 10.3389/fmed.2023.1239487 (PMC10470467; doi:10.3389/fmed.2023.1239487)

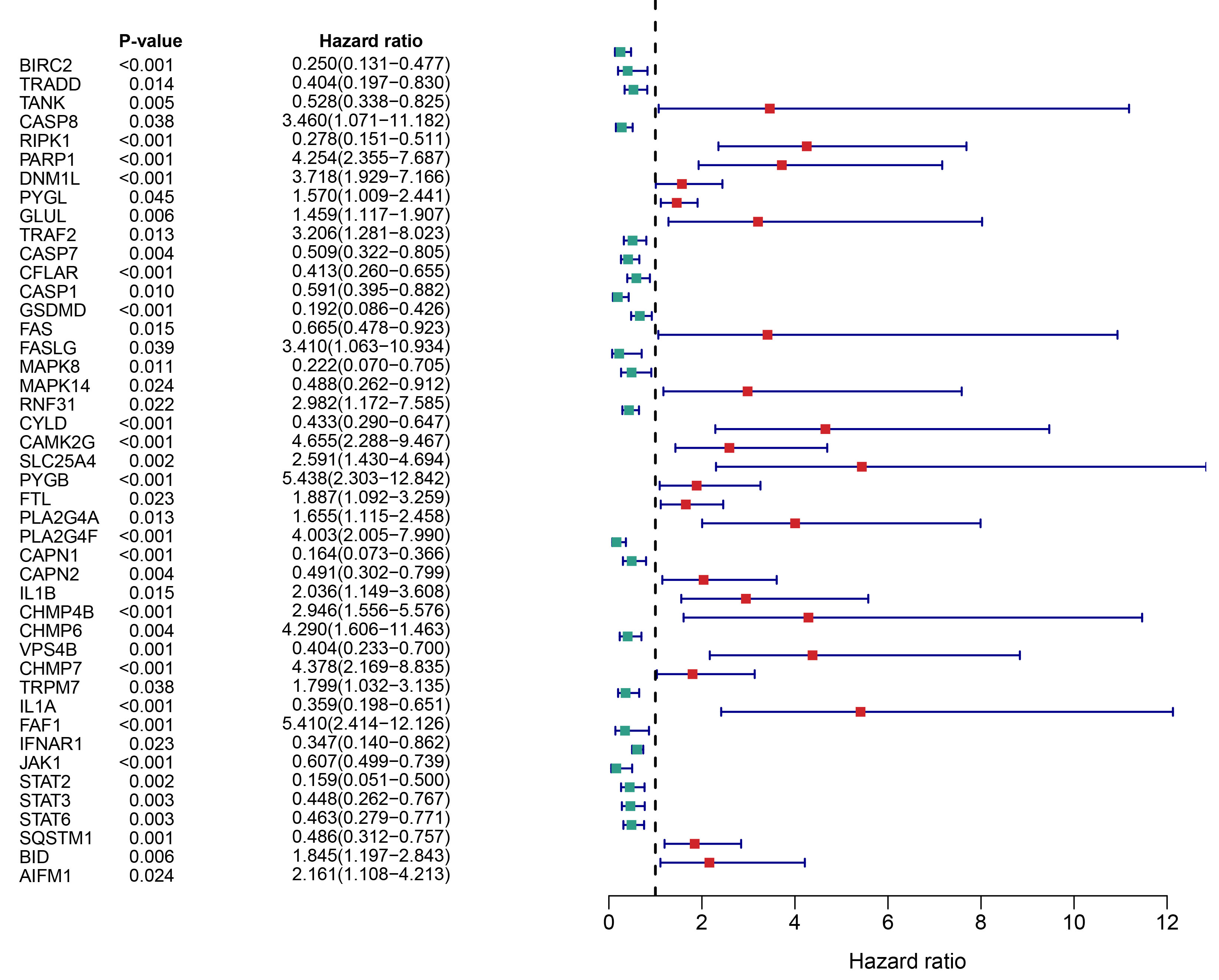

Supplement: Supplementary file 1 [file Data_Sheet_1.zip › supplementary material files/Supplementary Figure S1.jpg]

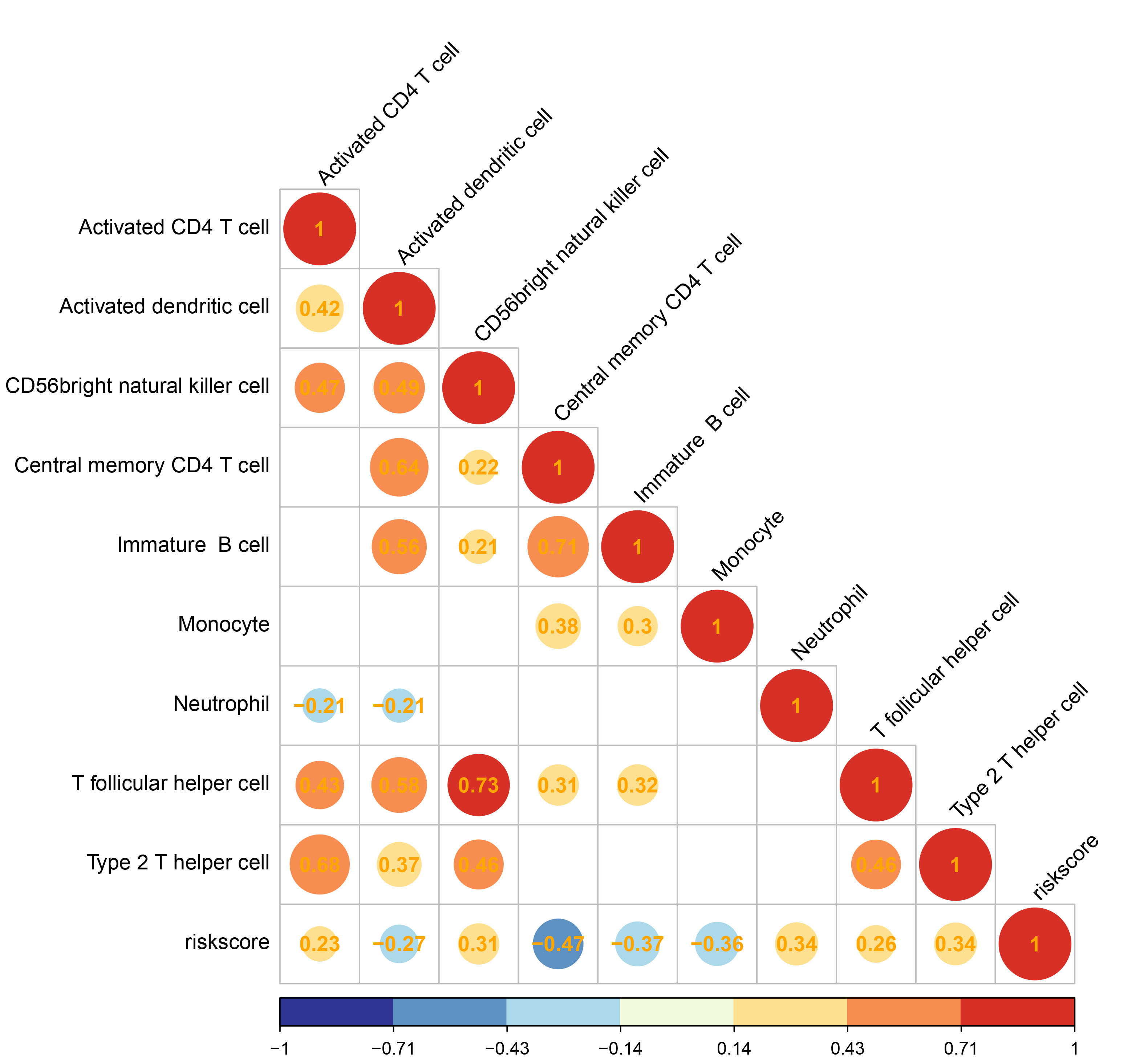

Supplement: Supplementary file 1 [file Data_Sheet_1.zip › supplementary material files/Supplementary Figure S2.jpg]
